# Copy Number Variation of Transposable Elements in *Thinopyrum intermedium* and Its Diploid Relative Species

**DOI:** 10.3390/plants9010015

**Published:** 2019-12-21

**Authors:** Mikhail G. Divashuk, Gennady I. Karlov, Pavel Yu. Kroupin

**Affiliations:** 1 Laboratory of Applied Genomics and Crop Breeding, All-Russia Research Institute of Agricultural Biotechnology, Moscow 127550, Russia; divashuk@gmail.com (M.G.D.);; 2 Centre for Molecular Biotechnology, Russian State Agrarian University-Timiryazev Agricultural Academy, Moscow 127550, Russia

**Keywords:** transposable elements, genome evolution, polyploidization, copy number variation, *Pseudoroegneria*, *Dasypyrum*, *Thinopyrum*

## Abstract

Diploid and polyploid wild species of *Triticeae* have complex relationships, and the understanding of their evolution and speciation could help to increase the usability of them in wheat breeding as a source of genetic diversity. The diploid species *Pseudoroegneria spicata* (St), *Thinopyrum bessarabicum* (J^b^), *Dasypyrum villosum* (V) derived from a hypothetical common ancestor are considered to be possible subgenome donors in hexaploid species *Th. intermedium* (J^r^J^vs^St, where indices r, v, and s stand for the partial relation to the genomes of *Secale*, *Dasypyrum*, and *Pseudoroegneria*, respectively). We quantified 10 families of transposable elements (TEs) in *P. spicata*, *Th. bessarabicum*, *D. villosum* (per one genome), and *Th. intermedium* (per one average subgenome) using the quantitative real time PCR assay and compared their abundance within the studied genomes as well as between them. *Sabrina* was the most abundant among all studied elements in *P. spicata*, *D. villosum*, and *Th. intermedium,* and among *Ty3/Gypsy* elements in all studied species. Among *Ty1/Copia* elements, *Angela-A* and *WIS-A* showed the highest and close abundance with the exception of *D. villosum*, and comprised the majority of all studied elements in *Th. bessarabicum*. *Sabrina*, *BAGY2,* and *Angela-A* showed similar abundance among diploids and in *Th. intermedium* hexaploid; *Latidu* and *Barbara* demonstrated sharp differences between diploid genomes. The relationships between genomes of *Triticeae* species based on the studied TE abundance and the role of TEs in speciation and polyploidization in the light of the current phylogenetic models is discussed.

## 1. Introduction

Transposable elements (TEs) are ubiquitous components of many studied eukaryotic genomes that are able to move around and proliferate within the host genome. The TE abundance ranges from 10% to 12% in the *Arabidopsis thaliana* and 70% to 80% in maize, barley, and wheat genomes [1,2,3,4,5,6,7,8]. TEs are categorized into the following two classes: class I (retrotransposons) that transpose via an element-encoded mRNA intermediate produced from a promoter in a long terminal repeat (LTR retrotransposons) or from an internal promoter (non-LTR retrotransposons) that is reverse-transcribed into DNA and integrated elsewhere in genome (“copy and paste”); and class II (DNA transposons) that transpose via DNA that is excised and reinserted into the host genome (“cut and paste”). Although the genomes of flowering plants have a rich collection of both TE classes, class I LTR retrotransposons are predominate in plant genomes, with prevailing families of *Ty1/Copia* and *Ty3/Gypsy* retrotransposons, determining the size of most plant genomes including monocots and dicots [9,10,11,12,13,14,15,16,17]. Among Class II TEs only five superfamilies have been found in plant genomes (*CACTA*, *Mutator*, PIF/Harbinger, *hAT*, and *Tc1/mariner*) [18,19].

TEs make a considerable contribution to evolutionary processes and speciation being one of the major forces in plant genome evolution [20,21,22,23]. Transposable elements affect the fitness of the host through altering expression or function of protein-coding genes. It may give rise to new alleles or even new gene formation and transcriptome variation, illegitimate recombination and chromosome breakage [24,25,26,27,28,29,30,31]. The hypothesis of ‘genetic shock’ suggests that interspecific hybridization may be accompanied by the proliferation or elimination of TEs leading to dramatic changes in a hybrid genome [32]. The dynamics of TEs, in particular, LTR retrotransposons, is supposed to be the main cause of plant genome size alterations on the evolutionary time scale [12,33,34,35,36,37]. Recent findings have shown that TEs are population-specific and apparently involved in population divergence and speciation at the diploid level [38,39,40]. 

In ancient allopolyploids and experimentally derived synthetic interspecific hybrids, the copy number of TEs is usually higher in comparison to their related diploid species increasing the plant genome size that is considered in some cases to be the result of ‘genome stresses’ [41,42,43,44,45]. However, depending on the TE family and plant species, the polyploidization may also be accompanied by the absence of changes in the TE copy number or even in the TE elimination [28,43,46,47,48]. Polyploidization and interspecific crosses have been shown to result in serious epigenetic changes that may involve modifying DNA methylation status of TE’s promoters [49,50]. A general trend seems to be the demethylation of TEs in newly formed allopolyploids that may be followed by returning to a methylation state similar to parental [51,52,53]. However, the observed methylation alternations depend on a unique TE, species genome and hybrid generation [28,42,50,53,54]. 

Among plant species, one of the highest TE abundance is observed in *Triticeae* species such as in oats (65%), rye (72%), wheat (80%), and barley (81%) [17,55,56,57]. Genetic and epigenetic dynamics of TEs have been thoroughly studied in wheat as compared with its genome donors (“evolutionary scale”) and in newly formed synthetic allopolyploids as compared with parental species (“revolutionary scale”) [28,52,53,54]. The study of the genomes of wheat relatives could help to find out the basis of *Triticeae* speciation and allopolyploidization, as well as the relationships between species including wild grasses. Among a huge variety of wild *Triticeae* species, closely related genomes St, J, and V are of particular interest as they can be found at different ploidy levels, namely, in diploids such as *Thinopyrum bessarabicum* (Savul. & Rayss) Á.Löve (2n = 2x = 14, J^b^), *Dasypyrum villosum* (L.) Coss. & Durieu ex P. Candargy (2n = 2x = 14, V), and *Pseudoroegneria spicata* (Pursh) Á.Löve 1980 (2n = 2x = 14, St), and presumably participated in the speciation of *Thinopyrum intermedium* (host) Barkworth & D.R. Dewey (2n = 6x = 42) with genomic composition J^r^J^vs^St, where J stands for the proximity to the genome of *Th. bessarabicum* (J^b^) or *Th. elongatum* (J^e^), and indices r, v and s demonstrate the presence of DNA repeats character for *Secale* (R genome), *Dasypyrum* (V genome) and *Pseudoroegneria* (St genome) species [58,59,60,61,62,63]. Additionally, the genomes of these species carry valuable genes that confer resistance to adverse environmental conditions (salinization, drought, frosts etc.), many of which have been successfully transferred to wheat via wide hybridization [64,65,66,67]. 

The study of TEs in wild *Triticeae* species could help to untangle the evolutionary relationship between St, J, and V genomes [68,69,70]. Although in all plant genomes, the majority of TEs is represented by LTR retrotransposons and the abundance of particular LTR families is highly variable among even closely related species [71]. The comparison of the TE’s abundance between the genomes of diploids and subgenomes in polyploids is of great interest as it may help to understand the speciation after the divergence from the common ancestor and the contribution of a particular genome in polyploid formation. Different molecular techniques ranging from conventional PCR to digital PCR as well as bioinformatic approaches have been applied to study TE’s polymorphisms that enable rapid and accurate identification of different TE’s insertions in the genome that helps to estimate genome modifications during evolution [72]. Recently, we demonstrated different abundance of *Ty3*/*Gypsy* centromeric retrotransposon on the individual chromosomes of particular subgenomes of *Th. intermedium* that may be the result of possible burst of this retrotransposon during allopolyploidization [73]. Moreover, the knowledge of transposable elements will provide the basis for the development of new PCR and cytogenetics markers that will facilitate the introgression of agronomically valuable traits into wheat genome [74,75]. 

The abundance of transposable elements (TEs) can be assessed using different approaches [72]. The computational analysis using NGS sequencing data have been successfully applied in *Triticum*, *Hordeum* and *Secale* studies [27,31,56,76]. However, such approach is limited due to the complex organization of TEs and the assembly problem caused by huge TE redundancy and may not be accurate for genomes that have not been successfully assembled [77]. The dot-blot and Southern hybridization are time and labor consuming procedures [78,79] while fluorescent in situ hybridization provide only qualitative comparison between genomes [79,80]. Real-time quantitative polymerase chain reaction (qPCR) is a powerful tool that enables an accurate estimation of copy number in genomes [28,39,42,52,73,81]. The data on relative TE abundance obtained using qPCR assay was shown to be very similar and comparable with data obtained from genome sequences analysis that demonstrates the efficiency of the qPCR approach [28,81].

The aim of this paper is to assess the interspecific variation of LTR retrotransposons and DNA transposon in polyploid *Th. intermedium* and its candidate progenitors, diploids *P. spicata*, *Th. bessarabicum*, and *D. villosum* using the qPCR assay. 

## 2. Results

We calculated the quantity of the TEs within genomes in *P. spicata* (St), *Th. bessarabicum* (J**^b^**), *D. villosum* (V), and *Th. intermedium* (J^r^J^vs^St) using normalization of the target sequences to the reference gene *VRN1*. It should be noted that there are differences in the size and nucleotide content of the fragments amplified from different TEs in one species. As a result, slightly different level of Eva Green^®^ binding with DNA is possible even if the abundance of different TEs is the same. And, as a consequence, slightly different level of fluorescence can be observed even at the same copy number. Therefore, we cannot consider this data as precise absolute values of TE copy number when comparing TE abundance within the studied genomes. However, since the amplicons of all the studied TEs have very similar size, we may reliably use this data to compare the abundance of different TEs between each other within the genomes of the studied species. 

Besides, we calculated the normalized relative TE’s quantity (NRQ) to compare genomes between each other. The divergence in TEs sequences among species during evolution and speciation could have affected both primer binding sites of the qPCR amplicon and sequences between them (inner regions). In the first case, nucleotide polymorphisms could result in primer mismatch and, eventually, imperfect amplification. This issue is addressed by the primer efficiency estimation and a corresponding coefficient is included in equation of the TE’s quantity (Table S1). In the case if dramatic changes in nucleotide sequences have happened in the inner region, then the melting temperature of the fragment of the same TE amplified at qPCR in different species would be significantly different. Additionally, if the TE divergence has occurred within species genome, two different melting temperatures would have observed. In our experiments, only primers the produced amplicons with single peak at the melting curve and similar melting temperature of the same TEs between different species were chosen (Figure S1, Table S2). Moreover, using a given primer pair we may amplify the pool of different variants of the same TE that are conservative in primer binding site and thus to trace the quantitative differences between genomes. 

### 2.1. TE Copy Number Variation within Genomes

**Figure 1:**
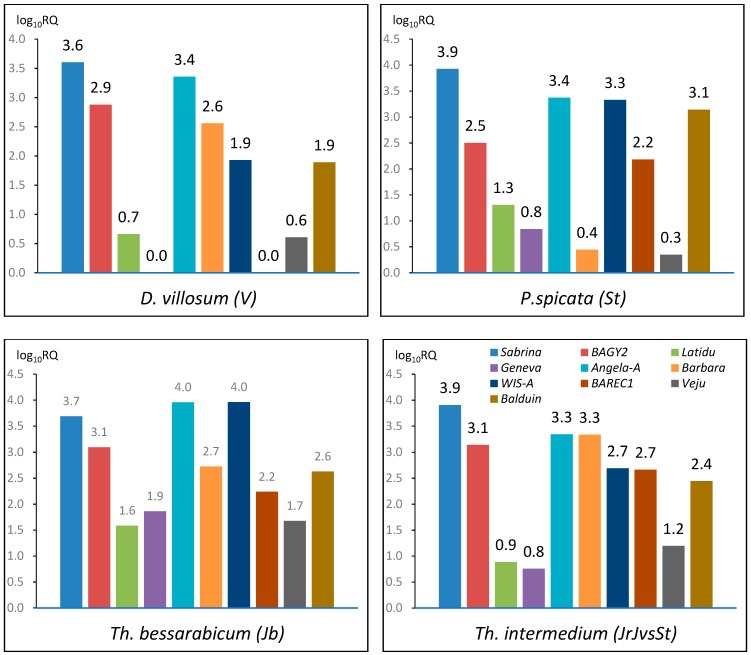
The decimal logarithm of relative quantity of TEs per one genome in *P. spicata* (St), *Th. bessarabicum* (J^b^), *D. villosum* (V), *Th. intermedium* (J^r^J^vs^St, the plot is for one average subgenome).

As a result of qPCR assay, the TEs within the studied genomes demonstrated high variability in their abundance (Figure 1, Table S3). In *P. spicata* (St), *Sabrina* showed the predominant majority among the studied TEs followed by *Angela-A* and *WIS-A*, whose abundance is ~4 times lower; *Balduin* is ~6 times lower. *BAGY2* and *BARE1C* showed moderate abundance, *Latidu*, *Geneva*, *Barbara* and *Veju* has low abundance (Figure S2a). In *Th. bessarabicum* (J^b^), the highest abundance was demonstrated similarly by *Angela-A* and *WIS-A* followed by *Sabrina* and *BAGY2* which content is ~2 and ~7 times lower, respectively. *Barbara*, *Balduin*, and *BARE1C* showed moderate abundance; *Geneva*, *Veju*, and *Latidu* have low abundance (Figure S2b). In *D. villosum* (V), the highest abundance was shown by *Sabrina*, while *Angela-A* and *BAGY2* content was 2 and 5 times lower, respectively. *WIS-A* has moderate copy number, *Veju*, *Barbara*, *Latidu*, *BARE1C* and *Balduin* have low abundance (Figure S2c). In *Th. intermedium* average subgenome, *Sabrina* is the most abundant element; *Angela-A*, *BAGY2* and *WIS-A* content is ~4 times lower. *Barbara*, *BARE1C* and *Balduin* have moderate content; *Veju*, *Latidu* and *Geneva* abundance is low (Figure S2d).

### 2.2. TE Copy Number Variation between Genomes

We compared the abundance of TEs (NRQ) between studied species (Table S4) and, in addition, compared it to TE content in *Aegilops* and *Triticum* species measured by Yaakov et al. 2013 [28]. To perform such comparison, *Ae. tauschii* was included in the experiment as the calibrator, as it was used in Yaakov et al. 2013 [28]. It also should be noted that we study the abundance of TEs using primers developed for wheat sequences. Therefore, we investigated TEs that more likely have derived from the common ancestor of the studied species and *Triticum*/*Aegilops* diploids. For the comparison with genomes of the diploid species, the results of the TE’s abundance in hexaploid *Th. intermedium* (J^r^J^vs^St) are described for one average diploid subgenome.

#### 2.2.1. *Sabrina*

The content of Ty-3 *Gypsy* LTR retrotransposon *Sabrina* in St, J^b^ and V genomes is close between each other (Figure S3). Moreover, it was comparable to the candidate donors of A, B and D genomes. The abundance in *Th. bessarabicum* (J^b^) and *D. villosum* (V) (0.7 and 0.5) is close to *Ae. speltoides* (S) (0.3–1.3), in *P. spicata* (St) (1.1) is close to *Ae. searsii* (S^s^) (1.0–1.6), *Ae. tauschii* (D) (1.0) and *T. urartu* (A^u^) (1.5). In one average subgenome of *Th. intermedium* (J^r^J^vs^St) *Sabrina* content is similar to the studied diploid species. 

#### 2.2.2. *BAGY2*

The content of *Gypsy* LTR retrotransposon *BAGY2* was also similar between genomes in the studied diploids (Figure S4). In *P. spicata* (St) (0.7) the *BAGY2* content was most close to its content observed in *Ae. longissima* (0.7) and was slightly lower than its content in *Ae. speltoides* (S) (0.8–1.4). The *BAGY2* abundance determined in our work in *D. villosum* (V) (1.6) and normalized to *Ae. tauschii* was in the range of variation of the abundance in *Ae. searsii* (S^s^) (1.5–3.9). Among diploid species studied in our work the highest abundance was revealed in *Th. bessarabicum* (J^b^) (2.4). In one average subgenome of *Th. intermedium* (J^r^J^vs^St) *BAGY2* content was close to that in St, J^b^ and V genomes. 

#### 2.2.3. *Latidu*

**Figure 2:**
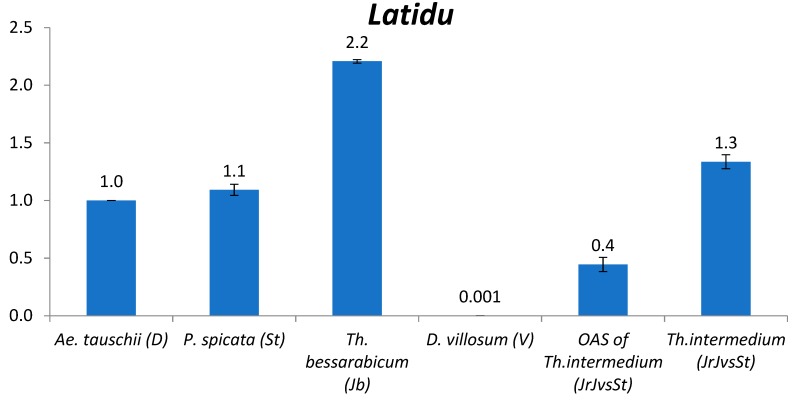
Relative quantification (compared to *Ae*. *tauschii*, which is taken as 1, see text) of *Gypsy* LTR retrotransposon *Latidu* in the following species: *Ae. tauschii*, *P. spicata*, *Th. bessarabicum*, *D. villosum*, one average subgenome (OAS) of *Th. intermedium*, and *Th. intermedium*. The numbers above chart bars are decimal logarithm of relative quantity of TEs; error bars show standard deviation.

The abundance of *Gypsy* LTR retrotransposon *Latidu* in St and J^b^ genomes was rather close; in V genome of *D. villosum* its abundance was three orders of magnitude lower than in St and J^b^ genomes (Figure 2). Additionally, the observed copy number in St and J^b^ was similar to that in the potential donors of B and D genomes of wheat: the abundance in *P. spicata* (St) (1.1) and *Th. bessarabicum* (J^b^) (2.2) was in the range of *Ae. speltoides* (S) (0.6–3.0) and *Ae. tauschii* (D) (1.0). In one average subgenome of *Th. intermedium* (J^r^J^vs^St) *Latidu* content was closer to that in St and J^b^ genomes.

#### 2.2.4. *Geneva*

**Figure 3:**
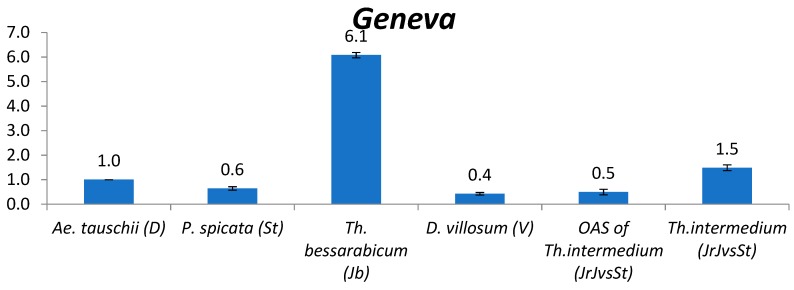
Relative quantification (compared to *Ae*. *tauschii*, set as 1, see text) of *Gypsy* LTR retrotransposon *Geneva* in the following species: *Ae. tauschii*, *P. spicata*, *Th. bessarabicum*, *D. villosum*, one average subgenome (OAS) of *Th. intermedium*, and *Th. intermedium*. The numbers above chart bars are decimal logarithm of relative quantity of TEs; error bars show standard deviation.

The content of *Gypsy* LTR retrotransposon *Geneva* is close between St and V genomes and ~10 times lower than in J^b^ (Figure 3). The *Geneva* copy number in St and V is similar to that in candidate donors of B, D and A genomes of wheat: *P. spicata* (St) and *D. villosum* (V) (0.6 and 0.4) are close to *Ae. searsii* (S^s^) and *T. urartu* (A^u^) (0.3–0.7). *Geneva* content in *Th. bessarabicum* (J^b^) (6.1) is approximately one order magnitude higher than in A, B, D, St and V. In one average subgenome of *Th. intermedium* (J^r^J^vs^St) *Geneva* content was close to St and V subgenomes. 

#### 2.2.5. *Angela-A*

The abundance of *Copia* LTR retrotransposon *Angela-A* abundance in the studied diploid and polyploid species was relatively close between each other (Figure S5). Only *Th. bessarabicum* (J^b^) (3.0) slightly prevailed in copy number both diploids and polyploids as calculated per one genome. *Th. intermedium* (J^r^J^vs^St) in its average genome has *Angela-A* abundance close to St, J^b^ and V genomes. The abundance of *Angela-A* in the studied genomes (1.1–3.0) was in the range (0.7–9.3) observed in diploids and polyploids studied in Yaakov et al. (2013).

#### 2.2.6. *Barbara*

**Figure 4:**
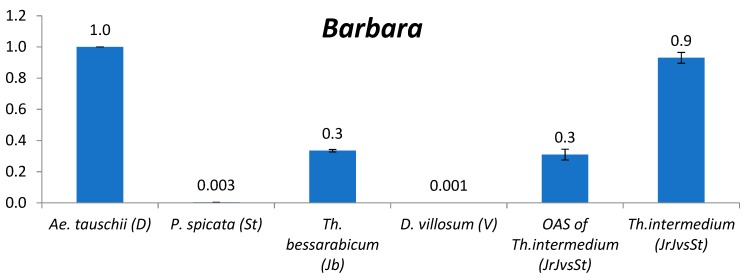
Relative quantification (compared to *Ae*. *tauschii*, set as 1, see text) of *Copia* LTR retrotransposon *Barbara* in the following species: *Ae. tauschii*, *P. spicata*, *Th. bessarabicum*, *D. villosum*, one average subgenome of *Th. intermedium*, and *Th. intermedium*. The numbers above chart bars are decimal logarithm of relative quantity of TEs; error bars show standard deviation.

*Barbara* demonstrated dramatic differences between the studied genomes: its content in *P. spicata* (St) and *D. villosum* (V) is two orders of magnitude lower than in J^b^ genome (Figure 4). The abundance of *Copia* LTR retrotransposon *Barbara* in St, J^b^ and V genomes (0.0006–0.3) is much lower than in *Aegilops* and *Triticum* diploids. One average subgenome of *Th. intermedium* (J^r^J^vs^St) has *Barbara* content two orders of magnitude higher than St and V and close to J^b^ genome. 

#### 2.2.7. *WIS-A*

We observed a similarity in the abundance of *Copia* LTR retrotransposon *WIS-A* between St (0.17) and J^b^ (0.7) genomes; V genome (0.03) showed the least abundance (Figure S6). *P. spicata* (St) (0.17) and *D. villosum* (V) (0.03) have lower *WIS-A* content than *Aegilops* and *Triticum* diploids (0.5–4.6); *Th. bessarabicum* (J^b^) (0.7) is in the range of its copy number variation in *Ae. speltoides* (S) (0.5–4.6). In one average subgenome of *Th. intermedium* (J^r^J^vs^St) *WIS-A* abundance (0.16) is in the range of diploid species *P. spicata* and *Th. bessarabicum*. 

#### 2.2.8. *BARE1C*

The content of *Copia* LTR retrotransposon in *P. spicata* (St), *Th. bessarabicum* (J^b^) and *D. villosum* (V) (0.2–0.3) was very much close between diploid species (Figure S7). The revealed range between St, V and J^b^ was lower than in *Aegilops* and *Triticum* diploids (1.0–10.8). The *BARE1C* content in one average subgenome of *Th. intermedium* (J^r^J^vs^St) is ~4 times its content in the studied diploids. 

#### 2.2.9. *Veju*

The abundance of *Copia*-like LTR retrotransposon *Veju* is close between St and V genomes; in J^b^ genome it is slightly higher (Figure S8). *P. spicata* (St) (0.1) and *D. villosum* (V) (0.2) have lower *Veju* abundance than *Aegilops* and *Triticum* diploids (1.0–10.4), *Th. bessarabicum* (J^b^) (1.3) is close to *Ae. tauschii* (D) (1.0). In one average subgenome of *Th. intermedium* the *Veju* content (0.4) is close to St and V genomes.

#### 2.2.10. *Balduin*

**Figure 5:**
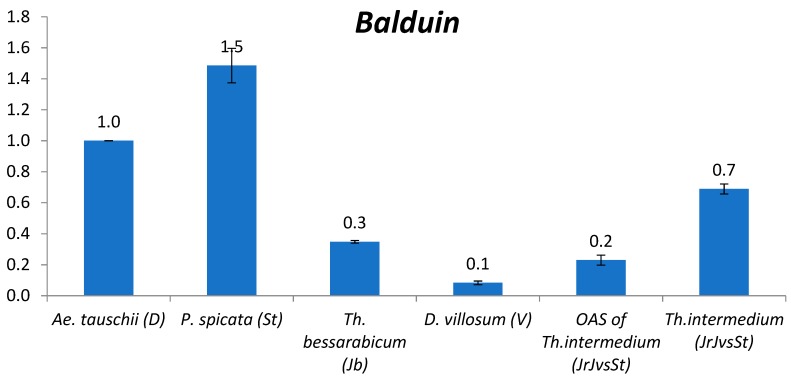
Relative quantification (compared to *Ae*. *tauschii*, set as 1, see text) of *CACTA* TIR transposon *Balduin* in the following species: *Ae. tauschii*, *P. spicata*, *Th. bessarabicum*, *D. villosum*, one average subgenome of *Th. intermedium*, and *Th. intermedium*. The numbers above chart bars are decimal logarithm of relative quantity of TEs; error bars show standard deviation.

The only revealed in our study DNA transposon is *CACTA* TIR transposon *Baldiun*. It demonstrated similarity between J^b^ (0.3) and V (0.1) genomes, the latter differed considerably from St genome (1.5) (Figure 5). *P. spicata* (St) (1.5) showed the highest abundance among studied diploids compared to *T. urartu* (A^u^); *Th. bessarabicum* (J^b^) (0.3) *Veju* content is close to *Ae. searsii* (S^s^); *D. villosum* (V) has the least quantity. In one average subgenome of *Th. intermedium* (J^r^J^vs^St), *Baldiun* content is one order of magnitude lower than is in *P. spicata* (St).

## 3. Discussion

In our qPCR experiments, we used the primers for TEs that were verified and proved that that they can be used to adequately assess TE abundance [28]. Therefore, if in our experiments with a given species, we observed positive amplification from a pair of the primers developed for a given *Triticum/Aegilops* TE, we may conclude that this particular TE had been present in the genome of the common ancestor of *Triticum/Aegilops* and the studied species. The difference in abundance between *Triticum/Aegilops* and our wheat related species evolved from the common ancestor could have occurred because of TE proliferation/elimination, methylation, polyploidization and other evolutionary factors. We here, for the first time, studied the abundance of nine LTR retrotransposons of *Gypsy* (*Latidu*, *Sabrina*, *BAGY2*, *Geneva*) and *Copia* (*Angela-A*, *Barbara*, *WIS-A*, *BARE1C*, and *Copia*-like *Veju*) superfamilies and one TIR transposon (*Balduin*) in *P. spicata* (St), *Th. bessarabicum* (J^b^), *D. villosum* (V), *Th. intermedium* (J^r^J^vs^St). Because of using *Ae. tauschii* as a calibrator we could compare the observed TE content in the studied species with published data for *Ae. searsii* (S^s^), *Ae. speltoides* (S), *Ae. sharonensis* (S^sh^), *Ae. longissima* (S^l^), *T. urartu* (A^u^), *Ae. tauschii* (D), *T. turgidum* ssp. *dicoccoides* (BA), *T. turgidum* ssp. *durum* (BA), and *T. aestivum* (BAD) [28]. 

### 3.1. The Comparison of TE Content within Genomes

In our experiments, the analysis of the relative quantities of four *Gypsy* retrotransposons families (*Sabrina*, *Latidu*, *BAGY2*, *Geneva*) demonstrated that together they comprise the majority of all studied TEs (and LTR retrotransposons, in particular), in all studied species with the exception for *Th. bessarabicum*. Of the TE components, Ty3/*Gypsy*, Ty3/*Copia* and *CACTA* are the most abundant in the published genomes of wheat, barley, *T. urartu*, and *Ae. tauschii*, *Agropyron cristatum* and *Secale cereale* [27,50,56,82]. In all studied species, *Sabrina* and *BAGY2* are highly abundant and dominates over other *Gypsy* LTR retroelements, while *BAGY2* is being less abundant than *Sabrina*. Moreover, non-autonomous *Gypsy* element *Sabrina* is the most abundant element among all studied elements in *P. spicata* and *D. villosum* andin polyploid *Th. intermedium*. Also, *Sabrina* is the second abundant element in many *Triticeae* species (*T. urartu*, *T. boeoticum*, *T. monococcum*, *Ae. tauschii*, *T. aestivum*, *H. spontaneum*, *H. vulgare*, *S. cereale*) [6,27,82]. *Gypsy* element *BAGY2* was found to be the third in abundance among the most abundant TEs in *Hordeum*, but has very low content in *Triticum* and *Aegilops* genomes [27,82]. In *Th. bessarabicum* and *D. villosum* the *BAGY2* content was comparable to the most abundant element and were fourth and third in the most abundant elements, respectively. In polyploid *Th. intermedium*, *BAGY2* was the fourth most abundant element. 

In our experiments, in all studied species *Copia* elements *Angela-A* and *WIS-A* showed very high and very close abundance, with exception for *D. villosum* (V), in which *WIS-A* content was ~6 times lower than *Angela-A*. In *Th. bessarabicum* (J^b^) *Angela-A* and *WIS-A* comprised the predominant majority; in *P. spicata* (St) and *Th. bessarabicum* (J^b^) *Angela-A* was the second abundant element. The *BARE1* clade in barley and *Angela-A*/*Wis* clade in wheat comprise approximately 10% of their genome and represent high-copy elements in *Triticeae* [6,83,84,85,86]. It should be noted that *WIS* and *Angela* are the wheat homologues of *BARE1* found in barley and in some studies, they are treated as the same family *BARE1* [6,27] or *Angela-A* [87]. In poylpoid *Th. intermedium*, *Angela-A* and *WIS-A* were found to be in the most abundant elements. *BARE1C* demonstrated rather moderate abundance in all studied species that may be explained by its specificity for *Hordeum* species. This clade is considered to have existed before the divergence of monocots and dicots, 150My ago [86]. Perhaps, *BARE1C* had been substituted by or transformed into its analogues *Angela-A* and *WIS-A* in the common ancestor of A, B, D, St, J^b^, and V genomes. *Barbara* showed from low to moderate abundance in the studied species; *Copia*-like element *Veju* demonstrated rather low abundance in all studied species.

### 3.2. The Comparison of TE Content between Genomes

The following patterns were found between St, J^b^, V, A^u^, S, and В genome diploids when the data on the TE abundance was combined with the phylogenetic tree based on the GBSSI gene sequences [88] (Figures S9–S11). *Sabrina*, *BAGY2*, and *Angela-A* demonstrated relative similarity between *Thinopyrum* and *Triticum*-related species that may indicate that the elements were inserted in their common ancestor and that the content of these elements was not changed dramatically since that time. *Latidu*, *Geneva*, *Barbara*, and *WIS-A* demonstrated dramatic differences between both *Thinopyrum* and *Triticum*-related species. This may be associated with their low content in their common ancestor and further multiple reinsertion and proliferation in particular genomes after their divergence. In general, the TE content in *Th. bessarabicum* was the most similar to *Triticum*/*Aegilops* diploids, especially, to *Ae. tauschii* that may be associated with their speciation from the common ancestor. 

The abundance of *Gypsy* LTR retrotransposons *Sabrina* and *BAGY2* in the studied diploids, hexaploid *Th. intermedium* and *Triticum*/*Aegilops* diploids were quite close (Figure S9). It may be the evidence of that these TE’s increased their abundance in the common ancestor and then did not change it significantly after the divergence of the considered species. *Sabrina* is probably an ancient element that inserted into common ancestor of *Triticeae* at least 9.65 million years ago (Mya) and then insertion events repeated multiple times [89,90]. *Sabrina*-like LTR pDbH12 was used to develop PCR and FISH markers to differentiate genomes V (*D. villosum*), V^b^ (*D.breviaristatum*), and J^vs^ (*Th. intermedium*) from other *Triticeae* genomes [60,91]. Among other members of *Gypsy* superfamily *Sabrina* demonstrated the lowest evolutionary dynamics [28]. We can suggest that all subsequent *Sabrina* proliferations that occurred in the genomes of *Triticeae* species were not so dramatic as compared to the first massive insertion event(s) in the common *Triticeae* ancestor. 

*Copia* LTR retrotransposons *Angela-A*, *WIS-A*, and *BARE1C* are clustered as one ancient clade in the published studies; they have reinserted in wheat and barley genomes for multiple times for more than 3 million years and show different copy number variation among *Triticeae* species [27,86]. *Angela-A* showed similar abundance among the studied diploids, hexaploid *Th. intermedium* as well as in *Triticum*/*Aegilops* diploids [28] that may indicate its evolutionary stability. 

*BARE1C* was shown to be much more abundant in *Secale* and *Hordeum* rather than in *Triticum*/*Aegilops* species [27]. In our studies, diploid *P. spicata*, *Th. bessarabicum*, and *D. villosum* demonstrated the *BARE1C* abundance even lower than in *Triticum*/*Aegilops* species (Figure S10). In polyploid *Th. intermedium*, *BARE1C* content was higher than in its relative diploids. Therefore, we may suppose that once inserted in the common ancestor of *Triticeae* it became later more abundant in *Hordeum* and *Secale* (H–R) and less abundant in *Triticum/Aegilops* and *Thinopyrum*-related species. However, it kept its evolutionary activity in *Triticum/Aegilops* species [28] and, perhaps, might have played a significant role at polyploidization in *Thinopyrum intermedium*. J^r^ subgenome of *Th. intermedium* and R genome of *Secale* share genomic sequences [63,92] that also might be associated with higher abundance of *BARE1C* in *Th. intermedium* compared to its related diploid species. 

*WIS-A* is supposed to have inserted for the first time in *Triticeae* ancestor at least 4.5 Mya and characterized by relatively low activity in wheat [28,89]. In our experiments, its abundance was lower than in *Triticum*/*Aegilops* species that may indicate its evolutionary elimination in this V-St branch of *Triticeae* speciation. Interestingly, Monte et al. (1995) clustered *Th. bessarabicum* with *Triticum* species based on *WIS 2-1A* sequences [93]; in our experiments, *Th. bessarabicum* demonstrated *WIS-A* abundance the most similar to *Aegilops tauschii* among other studied species (Figure S10). In the phylogenic studies, the J genome of *Thinopyrum* was shown to be closer to D genome of *Ae. tauschii* than St [88,94,95]. The similarity in TE content may be the possible reason for such closeness and the drive force for the recombination between these genomes [23]. That may explain why the majority of chromosomal rearrangements in wide hybrids occurs between J^r^ и J^vs^ genomes of *Th. intermedium* and D genome of wheat [96]. 

The abundance of *Copia* LTR retrotransposon *Barbara* and *Veju* in St and V genomes is lower than in *Aegilops* and *Triticum* diploids. *Barbara* and *Veju* are recent elements that character for *Triticum/Aegilops* species and are affected in early generations in experimentally derived hybrids [28,40,86,97]. In the J^b^ genome of *Th. bessarabicum*, *Veju* and *Barbara* abundance was the closest to D genome of *Ae. tauschii* among studied species that may explain the J^b^ and D genomes proximity and might be associated with their recent divergence from the common J^b^-D ancestor (Figure S10). The suggestions inferred from our results coincides with the phylogenetic tree based on the GBSSI sequences [88]. 

Sharp differences in *Latidu* and *Barbara* abundance (several orders of magnitude) and more slight differences in *Geneva, Veju*, and *Balduin* between St, J^b^, and V genome diploids (Figures S9–S11) could have favored a necessary level of difference between homeologous chromosomes of closely related subgenomes in newly formed *Thinopyrum* allopolyploids for relatively stable meiosis. In this case, the differences in certain TEs between putative contributors of subgenomes in *Thinopyrum* polyploids could have been a condition for efficient coexistence of them in one allopolyploid genome and functioning in one organism [23,73,98]. The comparison between the TE abundance in the St, J^b^, and V genome diploids and allopolyploid *Th. intermedium* revealed the following trends (Figure S12). *Latidu, Geneva, Angela-A*, and *Balduin* in their sum of contents in St, J^b^, and V exceeded their content in the whole genome of *Th. intermedium*. This may be explained by either the increase in their content in *Th. bessarabicum* (in case of *Latidu, Geneva, Angela-A*) and *P. spicata* (in case of *Balduin*) after the speciation of *Th. intermedium* or by their repression in *Th. intermedium* genome after allopolyploidization (Figure S12). In contrast, *Sabrina*, *BAGY2*, *Barbara*, and *BARE1C* in their sum of St, J^b^, and V was lower compared to the abundance observed in the whole genome of *Th. intermedium* that may be explained by their possible proliferation in intermedium wheatgrass after it evolved. Such changes in TE copy number are referred to as ‘genetic shock’ that is associated with the formation of new alleles, illegitimate recombination and chromosome rearrangements. However, the selection against such instability is relaxed due to the increase in gene copy number because of combining homeologs in one genome. As a result, polyploids with new benefit allelic variants and gene blocks may evolve that are more adapted to adverse environmental conditions [23]. This may be one of the reasons why polyploid *Th. intermedium* (2n = 42) and *Th. ponticum* (2n = 70) are widely used as sources of valuable genes for wheat improvement via wide hybridization [99]. 

In one average subgenome of *Th. intermedium* (J^r^J^vs^St) *Latidu* content was comparable to that in St and J^b^ genomes, whereas in V genome of *D. villosum* it was three orders of magnitude lower than in St and J. *Barbara* also showed close abundance between one average subgenome of *Th. intermedium* and J^b^ genome of *Th. bessarabicum*, while its content in St and V genomes was three orders of magnitude lower than in J^b^ genome. Apparently, the highest contribution to the *Latidu* abundance in *Th. intermedium* genome was made by the progenitors of *P. spicata* and *Th. bessarabicum*, while that of *Barbara* was made by the *Th. bessarabicum* progenitor. 

*Geneva* content was comparable between St and V genomes and one average subgenome of *Th. intermedium* (J^r^J^vs^St), whereas in J^b^ genome it was in several orders of magnitude higher; similar situation albeit with more slight differences is observed in *Angela-A*. On one hand, we may assume that these TEs proliferated in the *Th. bessarabicum* progenitor after the speciation of *Th. intermedium*. Alternatively, their content in J^b^ subgenome donor was much higher than in St and V progenitors as today, but in a newly formed *Th. intermedium* genome it was suppressed and eventually eliminated (Figure S12).

## 4. Conclusions

In conclusion, in this research, we have performed an analysis of the relative quantity of 9 TE families representing retrotransposons and 1 TE family of DNA transposon in hexapoloid *Th. intermedium* (J^r^J^vs^St) and its hypothetical subgenome contributors, three related diploids *P. spicata* (St), *Th. bessarabicum* (J^b^), and *D. villosum* (V) and compared its abundance with *Triticum/Aegilops* species. We discovered, the *Sabrina*, *BAGY2,* and *Angela-A* demonstrated similar abundance in St, J^b^, V and *Triticum/Aegilops* genomes that may indicate that its massive proliferation in their putative common ancestor. *BARE1C* showed close abundance between St, J^b^, V and lower abundance than in B, A and D subgenome contributors that may be associated with their elimination in the St-Jb-V possible common ancestor. *Latidu* and *Barbara* showed sharp differences and *Geneva, Veju*, *Balduin,* and *WIS-A* showed slighter differences between St, J^b^, and V that may indicate that their evolution more probably associated with *P. spicata*, *Th. bessarabicum* and *D. villosum* speciation rather than with their common ancestor. Based on the close abundance we may suggest that *Latidu* contribution in *Th. intermedium* genome was made by the progenitors of *P. spicata* and *Th. bessarabicum*, while that of *Barbara* was made by the *Th. bessarabicum* progenitor. Higher content *BARE1C* in *Th. intermedium* may be associated with J^r^ relationship to R genome in *Secale cereale*, where this TE is abundant; *WIS-A, Veju* and *Barbara* content in *Th. bessarabicum* and *Th. intermedium* was close to *Ae. tauschii* that may be associated with the closeness of J^b^, J^r^ and J^vs^ to D genome. 

## 5. Materials and Methods 

### 5.1. Plant Material

The plants were obtained from Germplasm Research International Network: *P. spicata* (PI 635993), *Th. bessarabicum* (PI 531711), *D. villosum* (W6 21717), *Th. intermedium* (PI 401200), and *Ae. tauschii* (CIae 3). DNA was extracted according to the protocol in [100] from the leaves of adult plants growing in the greenhouse. 

### 5.2. Quantitative PCR 

qPCR assay was performed according to Yaakov et al. (2013) [28]. The abundance of the studied TEs was estimated for target species, *P. spicata*, *Th. bessarabicum*, *D. villosum*, and *Th. intermedium*. To compare TE’s abundance between the target species, we used *Ae. tauschii* as a calibrator, since this species demonstrated the positive amplification of all the studied TEs in [28].

Each independent experiment was repeated minimum three times. In each experiment the real-time qPCR amplification was run in triplicate reactions (technical replicates) on the LightCycler^®^96 instrument (Roche Diagnostics, Mannheim, Germany). Each PCR mix consisted of 2.5 μL of reaction mix containing Eva Green^®^ (Syntol LTD, Hamilton, New Zeland), serially diluted DNA template (10, 2, 0.4 and 0.08 ng), 1.0 μL of each forward and reverse primer (10 pM/μL). We quantified the following transposable elements: LTR retrotransposons of *Ty3/Gypsy* (*Latidu*, *Sabrina*, *BAGY2*, *Geneva*) and *Ty1/Copia* (*Angela-A*, *Barbara*, *WIS-A*, *BARE1C*, and *Copia*-like *Veju*) superfamilies; TIR transposon (*Balduin*); for qPCR we used the primers designed in Yaakov et al. (2013) (Table S5). We compared each reaction to amplification of the *VRN1* gene (reference gene), as this gene is found in one copy in wheat genome and other studied genome as well [52]. As a result of qPCR, the value of the threshold cycle (Cq) was registered for each reaction. 

The efficiency of the PCR reactions (E) was determined by a standard curve through serial dilutions for the primer pairs for reference gene and target sequences for each species and calculated using the LightCycler^®^ 96 software and can be found in Table S1. The further PCRs for the calculations of TE abundance were done using primers with efficiency in the range of 1.8–2.1. If the primer efficiency values were out of this range than the data were excluded from the calculations. 

For validation of the absence of additional PCR products except for the amplified from the template of TE’s DNA, we performed melting curve protocol using LightCycler^®^ 96 instrument immediately after amplifications and ran the products of the PCR reaction on agarose gel. Only primers that produced a single melting curve peak with similar melting temperature of the amplicon and a single electrophoregram band were included in the following study. Examples of melting curves can be found in Figure S1. Melting temperatures of the amplicons can be found in Table S2.

### 5.3. TE’s Copy Number Calculation

In each species, the relative quantity of each TE (RQ) compared to the reference gene in the genome of a diploid species or in one average subgenome of polyploid *Th. intermedium* was calculated using the LightCycler^®^96 software [101] and used for the comparisons between the TE’s quantities inside a given species genome (or average subgenome). The normalized relative quantity (NRQ) and standard deviation was calculated using the following equation [28,102]:

$$\mathrm{NRQ}=N\cdot \left(\frac{E_{\mathrm{Ref}}{}^{{\mathrm{Cq}}_{\mathrm{target}}}}{E_{\mathrm{Tar}}{}^{{\mathrm{Cq}}_{\mathrm{target}}}}:\frac{E_{\mathrm{Ref}}{}^{{\mathrm{Cq}}_{\mathrm{calibrator}}}}{E_{\mathrm{Tar}}{}^{{\mathrm{Cq}}_{\mathrm{calibrator}}}}\right),$$

where “*E*_Tar_” and “*E*_Ref_” stand for the efficiency of the primers for the studied target transposable elements and the reference gene (*VRN1*), respectively; “Cq_target_” and Cq_calibrator_” stand for qPCR threshold cycles for the target species (*P. spicata*, *Th. bessarabicum*, *D. villosum*, and *Th. intermedium*) and the calibrator (*Ae. tauschii*), respectively. A part of the equation in parenthesis results in the quantification of TE’s abundance per one genome for a diploid or one average subgenome for a polyploid; for a calculation of the TE’s abundance in a whole haploid genome, it is multiplied by N, which is equivalent to the ploidy level: N = 1 for a diploid species, *P. spicata*, *Th. bessarabicum*, and *D. villosum*, and for one average diploid subgenome of hexpaloid *Th. intermedium*; N = 3 for the estimation of TE’s abundance in the whole genome of hexpaloid *Th. intermedium*. For the comparisons between diploids and hexaploid *Th. intermedium* we calculated the NRQ value for an average subgenome of *Th. intermedium* (N = 1), as the amplification in PCR occurs from all three subgenomes in hexaploid and it cannot be found using qPCR from which subgenome in particular. Thus, NRQ in *Ae. tauschii* is equal to 1. NRQ values were used for comparing the TE’s quantities between the studied genomes. The mean NRQ value and standard deviations were calculated as described in [103]. 

## Supplementary Materials

The following are available online at https://www.mdpi.com/2223-7747/9/1/15/s1, Figure S1: Examples of melting curves for the fragments amplified in qPCR using primers for *Geneva*, *WIS-A,* and *VRN1* (as a reference gene) in *P. spicata, Th. bessarabicum, D. villosum,* and *Ae. tauschii*, Figure S2: Relative quantity of TEs per one genome in the following species: a) *P. spicata* (St), b) *Th. bessarabicum* (J^b^), c) *D. villosum* (V), d) *Th. intermedium* (J^r^J^vs^St), Figure S3: Normalized relative quantity (compared to *Ae*. *tauschii*, set as 1, see text) of *Gypsy* LTR retrotransposon *Sabrina* in the following species: *Ae. tauschii*, *P. spicata*, *Th. bessarabicum*, *D. villosum*, one average subgenome (OAS) of *Th. intermedium*, and *Th. intermedium*. The numbers above chart bars are decimal logarithm of relative quantity of TEs; error bars show standard deviation, Figure S4: Normalized relative quantity (compared to *Ae*. *tauschii*, set as 1, see text) of *Gypsy* LTR retrotransposon *BAGY2* in the following species: *Ae. tauschii*, *P. spicata*, *Th. bessarabicum*, *D. villosum*, one average subgenome (OAS) of *Th. intermedium*, and *Th. intermedium*. The numbers above chart bars are decimal logarithm of relative quantity of TEs; error bars show standard deviation, Figure S5: Normalized relative quantity (compared to *Ae*. *tauschii*, set as 1, see text) of *Copia* LTR retrotransposon *Angela-A* in the following species: *Ae. tauschii*, *P. spicata*, *Th. bessarabicum*, *D. villosum*, one average subgenome of *Th. intermedium*, and *Th. intermedium*. The numbers above chart bars are decimal logarithm of relative quantity of TEs; error bars show standard deviation, Figure S6: Normalized relative quantity (compared to *Ae*. *tauschii*, set as 1, see text) of *Copia* LTR retrotransposon *WIS-A* in the following species: *Ae. tauschii*, *P. spicata*, *Th. bessarabicum*, *D. villosum*, one average subgenome of *Th. intermedium*, and *Th. intermedium*. The numbers above chart bars are decimal logarithm of relative quantity of TEs; error bars show standard deviation, Figure S7: Normalized relative quantity (compared to *Ae*. *tauschii*, set as 1, see text) of *Copia* LTR retrotransposon *BARE1C* in the following species: *Ae. tauschii*, *P. spicata*, *Th. bessarabicum*, *D. villosum*, one average subgenome of *Th. intermedium*, and *Th. intermedium*. The numbers above chart bars are decimal logarithm of relative quantity of TEs; error bars show standard deviation, Figure S8: Normalized relative quantity (compared to *Ae*. *tauschii*, set as 1, see text) of *Copia*-like LTR retrotransposon *Veju* in the following species: *Ae. tauschii*, *P. spicata*, *Th. bessarabicum*, *D. villosum*, one average subgenome of *Th. intermedium*, and *Th. intermedium*. The numbers above chart bars are decimal logarithm of relative quantity of TEs; error bars show standard deviation, Figure S9: Normalized relative quantity (NRQ, compared to *Ae*. *tauschii* (D), set as 1, see text) of *Gypsy* LTR retrotransposons in the following species: *T. urartu* (A^u^), *Ae. speltoides* (S), *Ae*. *tauschii* (D) (Yaakov et al., 2013); *P. spicata* (St), *Th. bessarabicum* (J^b^), *D. villosum* (V). The area of the circle is equal to the NRQ of each transposable element, Figure S10: Normalized relative quantity (NRQ, compared to *Ae*. *tauschii* (D), set as 1, see text) of *Copia* LTR retrotransposons in the following species: *T. urartu* (A^u^), *Ae. speltoides* (S), *Ae*. *tauschii* (D) (Yaakov et al., 2013); *P. spicata* (St), *Th. bessarabicum* (J^b^), *D. villosum* (V). The area of the circle is equal to the NRQ of each transposable element, Figure S11: Normalized relative quantity (NRQ, compared to *Ae*. *tauschii* (D), set as 1, see text) of DNA transposon *Balduin* in the following species: *T. urartu* (A^u^), *Ae. speltoides* (S), *Ae*. *tauschii* (D) (Yaakov et al., 2013); *P. spicata* (St), *Th. bessarabicum* (J^b^), *D. villosum* (V). The area of the circle is equal to the NRQ of each transposable element, Figure S12: Normalized relative quantity (compared to *Ae*. *tauschii* (D), set as 1, see text) of the studied mobile elements in the following species: *P. spicata* (St), *Th. bessarabicum* (J^b^), *D. villosum* (V), *Th. intermedium* (J^r^J^vs^St, per whole allopolyploid genome), Table S1: The efficiency of the primers used in qPCR experiments with serial dilutions of DNA template (10, 2, 0.4 and 0.08 ng per 25 μL of PCR mix) for each primer pair as calculated using LightCycler96 software; Table S2: Melting temperature of the fragments amplified in qPCR in *P. spicata*, *Th. bessarabicum*, *D. villosum*, *Th. intermedium*; Table S3: The relative quantity (RQ) of nine LTR retrotransposons and one TIR transposon in *P. spicata*, *Th. bessarabicum*, *D. villosum*, *Th. intermedium* (per one average subgenome), *Th. ponticum*. The RQ is calculated per one average genome of each species, Table S4: Normalized relative quantity (NRQ, compared to *Ae*. *taushii*, set as 1) of nine LTR retrotransposons and one TIR transposon in *P. spicata*, *Th. bessarabicum*, *D. villosum*, *Th. intermedium*. In *Th. intermedium*, NRQ is calculated per one average subgenome (×1) and per total genome (×3); Table S5: Primer sequences for the transposable elements and reference gene *VRN1* (Yaakov et al., 2013; Kraitshtein et al., 2010).
