# Supplementary material for: Copy Number Variation of Transposable Elements in Thinopyrum intermedium and Its Diploid Relative Species
Source: Plants (Basel). 2019 Dec 21;9(1):15. doi: 10.3390/plants9010015 (PMC7020174; doi:10.3390/plants9010015)
Supplement: Supplementary file 1 [file plants-09-00015-s001.zip › plants-620983-Supplementary/Figure S1.pdf]

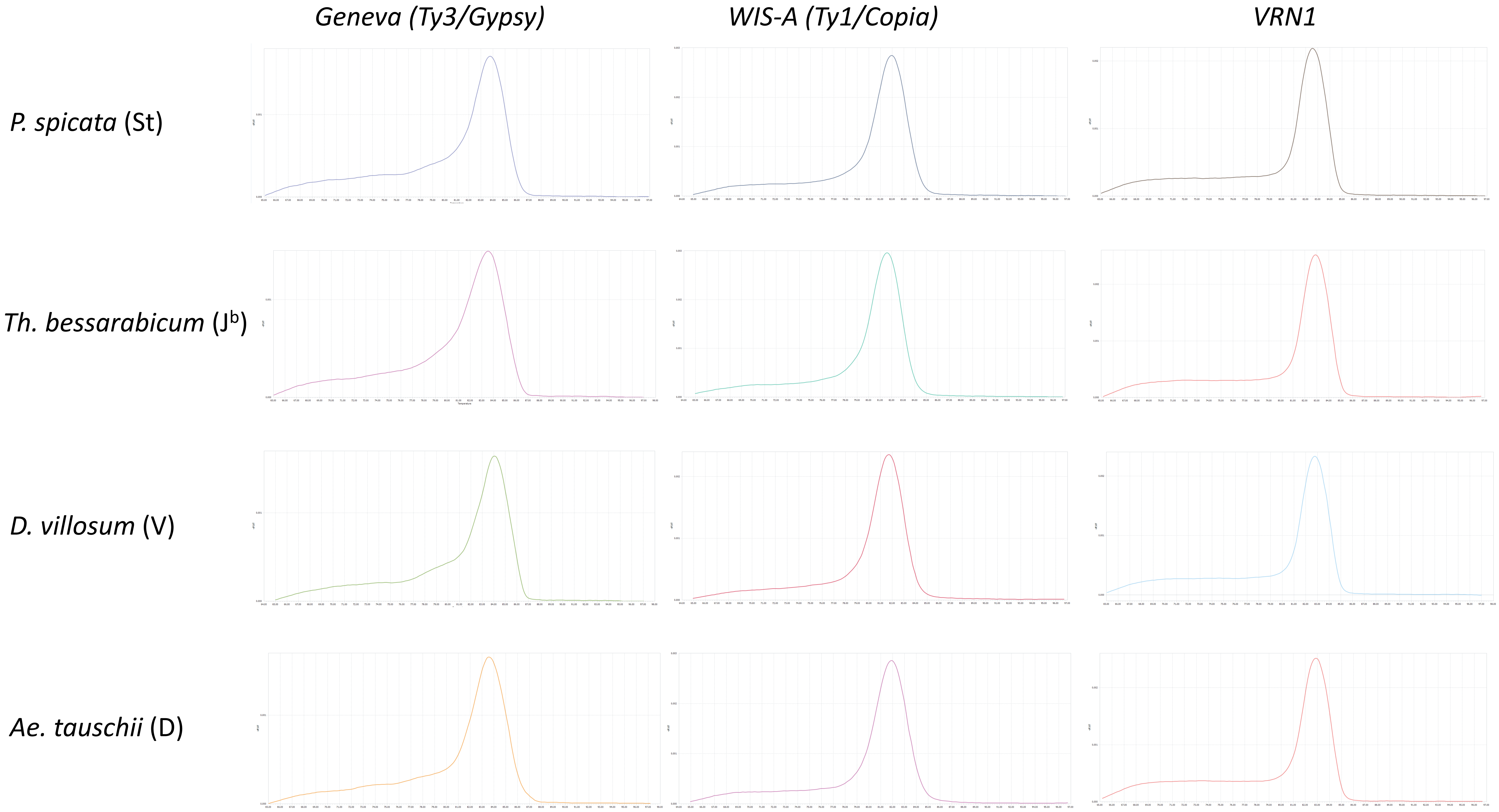

Figure S1. Examples of melting curves for the fragments amplified in qPCR using primers for *Geneva*, *WIS-A*, and *VRN1* (as a reference gene) in *P. spicata*, *Th. bessarabicum*, *D. villosum*, and *Ae. tauschii*.
