# Supplementary material for: Copy Number Variation of Transposable Elements in Thinopyrum intermedium and Its Diploid Relative Species
Source: Plants (Basel). 2019 Dec 21;9(1):15. doi: 10.3390/plants9010015 (PMC7020174; doi:10.3390/plants9010015)
Supplement: Supplementary file 1 [file plants-09-00015-s001.zip › plants-620983-Supplementary/Figure S2-S8.docx]

Copy Number Variation of Transposable Elements in *Thinopyrum intermedium* and Its Diploid Relative Species

Mikhail G. Divashuk ^1,2^, Gennady I. Karlov ^1,2^, Pavel Yu. Kroupin ^1,2,*^

^1^ Laboratory of Applied Genomics and Crop Breeding, All-Russia Research Institute of Agricultural Biotechnology, Timiryazevskaya st. 42 Moscow 127550 Russia; divashuk@gmail.com (M.G.D.), karlov@iab.ac.ru (G.I.K.), pavelkroupin1985@gmail.com (P.Yu.K.)

^2^ Centre for Molecular Biotechnology, Russian State Agrarian University-Timiryazev Agricultural Academy, Timiryazevskaya str. 49 Moscow 127550 Russia, divashuk@gmail.com (M.G.D.)

***** Correspondence: [pavelkroupin1985@gmail.com](mailto:pavelkroupin1985@gmail.com)

Supplementary material File 1 contains the following material: Figure S1, diagrams demonstrating the relative quantity of transposable elements per one genome in the studied species; Figures S2-S7, diagrams demonstrating the normalized relative quantity of transposable elements in the studied species

**Figure S2.** Relative quantity of TEs per one genome in the following species: a) *P. spicata* (St), b) *Th. bessarabicum* (J^b^), c) *D. villosum* (V), d) *Th. intermedium* (J^r^J^vs^St).

**Figure S3.** Normalized relative quantity (compared to *Ae*. *tauschii*, set as 1, see text) of *Gypsy* LTR retrotransposon *Sabrina* in the following species: *Ae. tauschii*, *P. spicata*, *Th. bessarabicum*, *D. villosum*, one average subgenome (OAS) of *Th. intermedium*, and *Th. intermedium*. The numbers above chart bars are decimal logarithm of relative quantity of TEs; error bars show standard deviation.

**Figure S4.** Normalized relative quantity (compared to *Ae*. *tauschii*, set as 1, see text) of *Gypsy* LTR retrotransposon *BAGY2* in the following species: *Ae. tauschii*, *P. spicata*, *Th. bessarabicum*, *D. villosum*, one average subgenome (OAS) of *Th. intermedium*, and *Th. intermedium*. The numbers above chart bars are decimal logarithm of relative quantity of TEs; error bars show standard deviation.

**Figure S5.** Normalized relative quantity (compared to *Ae*. *tauschii*, set as 1, see text) of *Copia* LTR retrotransposon *Angela-A* in the following species: *Ae. tauschii*, *P. spicata*, *Th. bessarabicum*, *D. villosum*, one average subgenome of *Th. intermedium*, and *Th. intermedium*. The numbers above chart bars are decimal logarithm of relative quantity of TEs; error bars show standard deviation.

**Figure S6.** Normalized relative quantity (compared to *Ae*. *tauschii*, set as 1, see text) of *Copia* LTR retrotransposon *WIS-A* in the following species: *Ae. tauschii*, *P. spicata*, *Th. bessarabicum*, *D. villosum*, one average subgenome of *Th. intermedium*, and *Th. intermedium*. The numbers above chart bars are decimal logarithm of relative quantity of TEs; error bars show standard deviation.

**Figure S7.** Normalized relative quantity (compared to *Ae*. *tauschii*, set as 1, see text) of *Copia* LTR retrotransposon *BARE1C* in the following species: *Ae. tauschii*, *P. spicata*, *Th. bessarabicum*, *D. villosum*, one average subgenome of *Th. intermedium*, and *Th. intermedium*. The numbers above chart bars are decimal logarithm of relative quantity of TEs; error bars show standard deviation.

**Figure S8.** Normalized relative quantity (compared to *Ae*. *tauschii*, set as 1, see text) of *Copia*-like LTR retrotransposon *Veju* in the following species: *Ae. tauschii*, *P. spicata*, *Th. bessarabicum*, *D. villosum*, one average subgenome of *Th. intermedium*, and *Th. intermedium*. The numbers above chart bars are decimal logarithm of relative quantity of TEs; error bars show standard deviation.
