# Supplementary material for: Copy Number Variation of Transposable Elements in Thinopyrum intermedium and Its Diploid Relative Species
Source: Plants (Basel). 2019 Dec 21;9(1):15. doi: 10.3390/plants9010015 (PMC7020174; doi:10.3390/plants9010015)
Supplement: Supplementary file 1 [file plants-09-00015-s001.zip › plants-620983-Supplementary/Figures S9-S12.pdf]

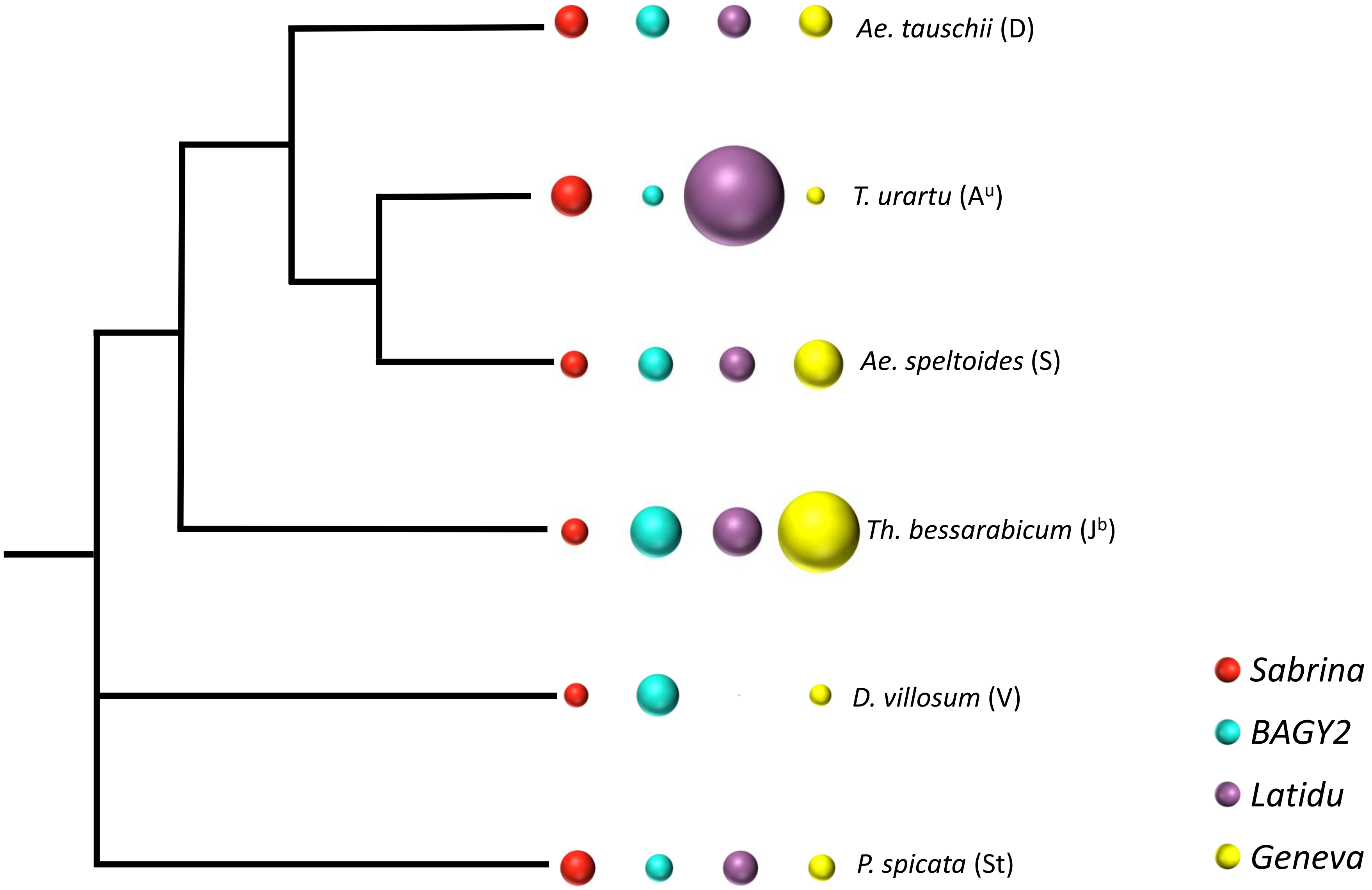

Figure S9. Normalized relative quantity (NRQ, compared to *Ae. tauschii* (D), set as 1, see text) of *Gypsy* LTR retrotransposons in the following species: *T. urartu* (A^u), *Ae. speltoides* (S), *Ae. tauschii* (D) (Yaakov et al., 2013); *P. spicata* (St), *Th. bessarabicum* (J^b), *D. villosum* (V). The area of the circle is equal to the NRQ of each transposable element.

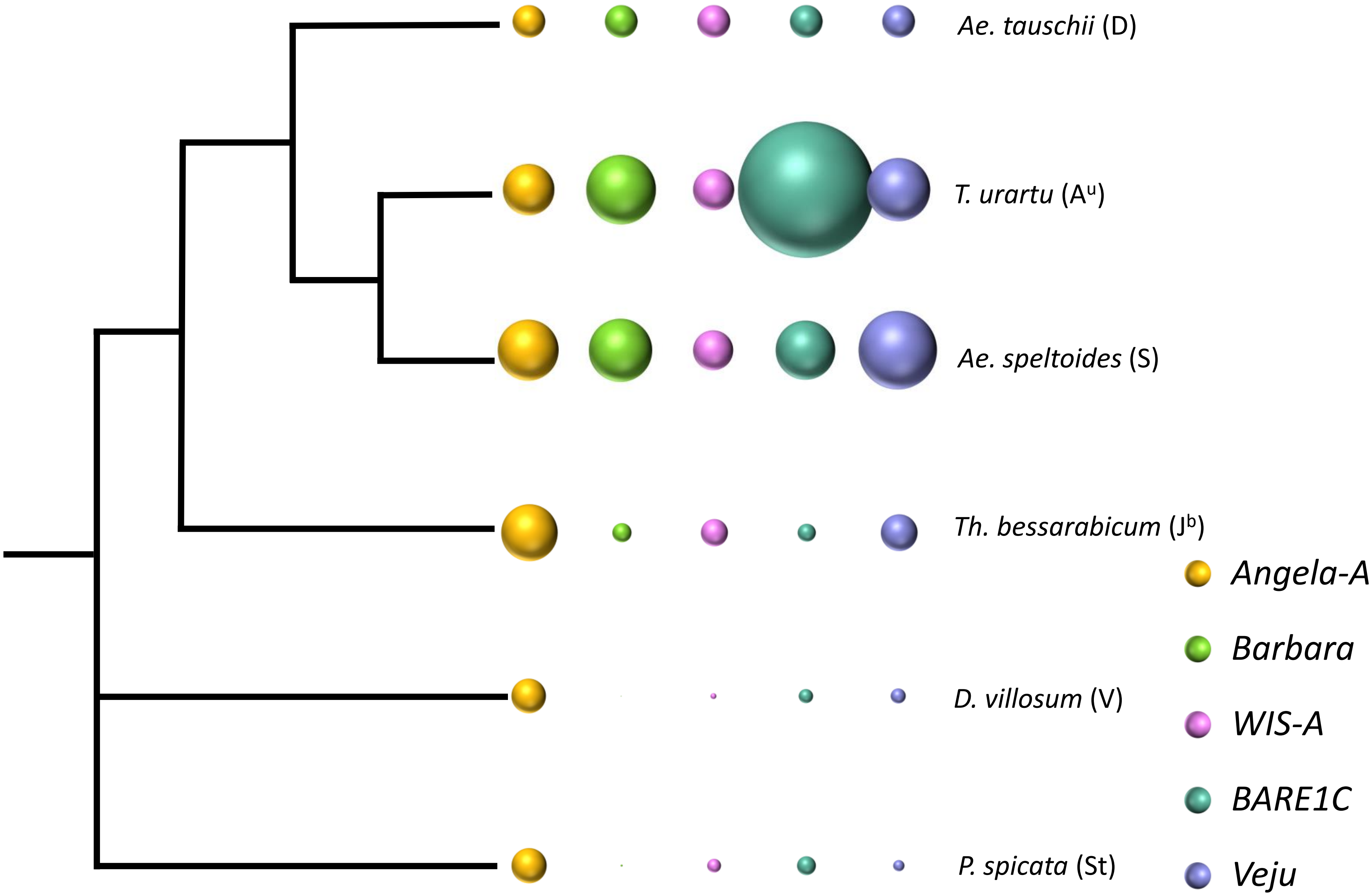

Figure S10. Normalized relative quantity (NRQ, compared to *Ae. tauschii* (D), set as 1, see text) of *Copia* LTR retrotransposons in the following species: *T. urartu* (A^u), *Ae. speltoides* (S), *Ae. tauschii* (D) (Yaakov et al., 2013); *P. spicata* (St), *Th. bessarabicum* (J^b), *D. villosum* (V). The area of the circle is equal to the NRQ of each transposable element.

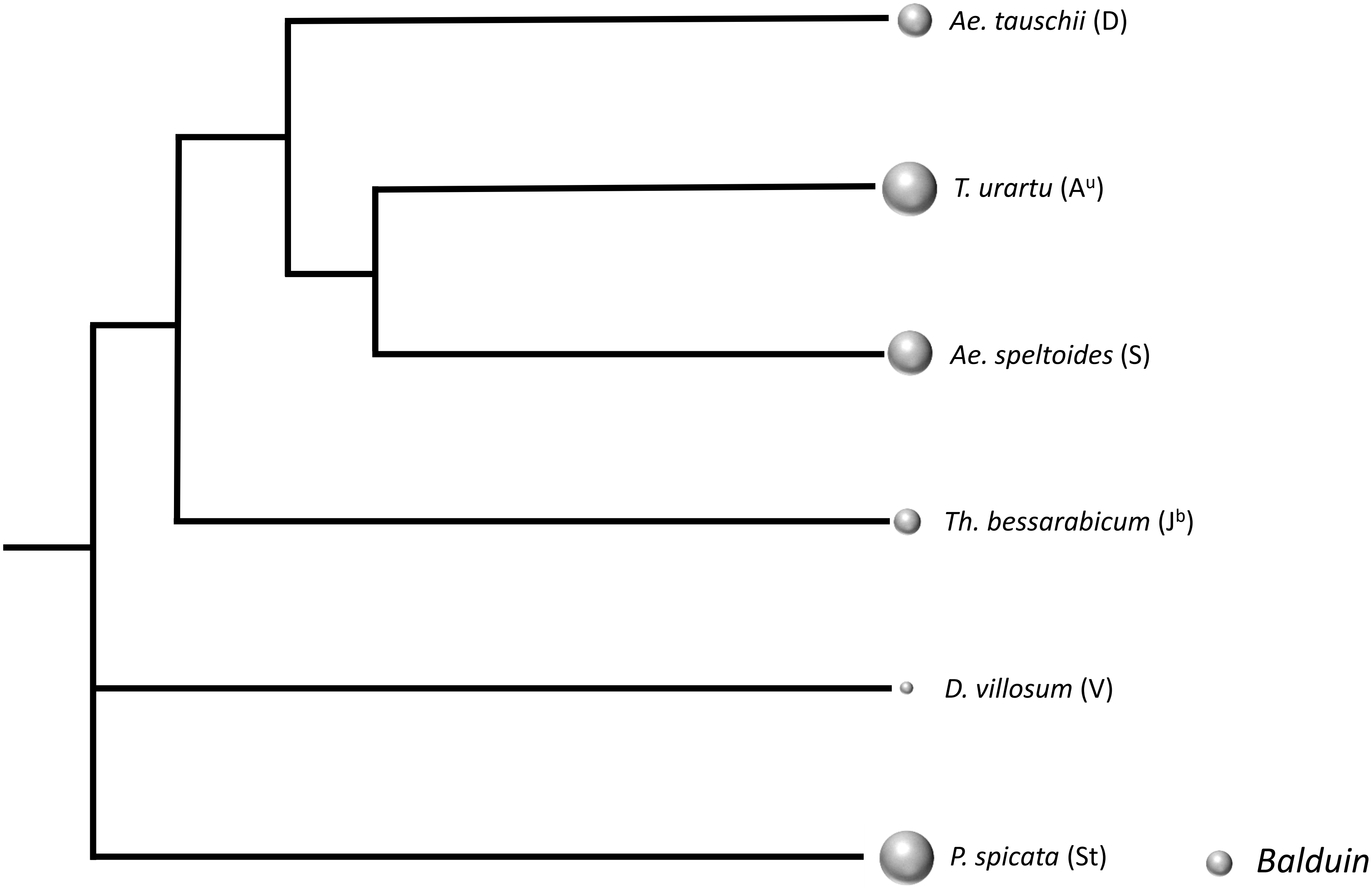

Figure S11. Normalized relative quantity (NRQ, compared to *Ae. tauschii* (D), set as 1, see text) of DNA transposon *Balduin* in the following species: *T. urartu* (A^u), *Ae. speltoides* (S), *Ae. tauschii* (D) (Yaakov et al., 2013); *P. spicata* (St), *Th. bessarabicum* (J^b), *D. villosum* (V). The area of the circle is equal to the NRQ of each transposable element.

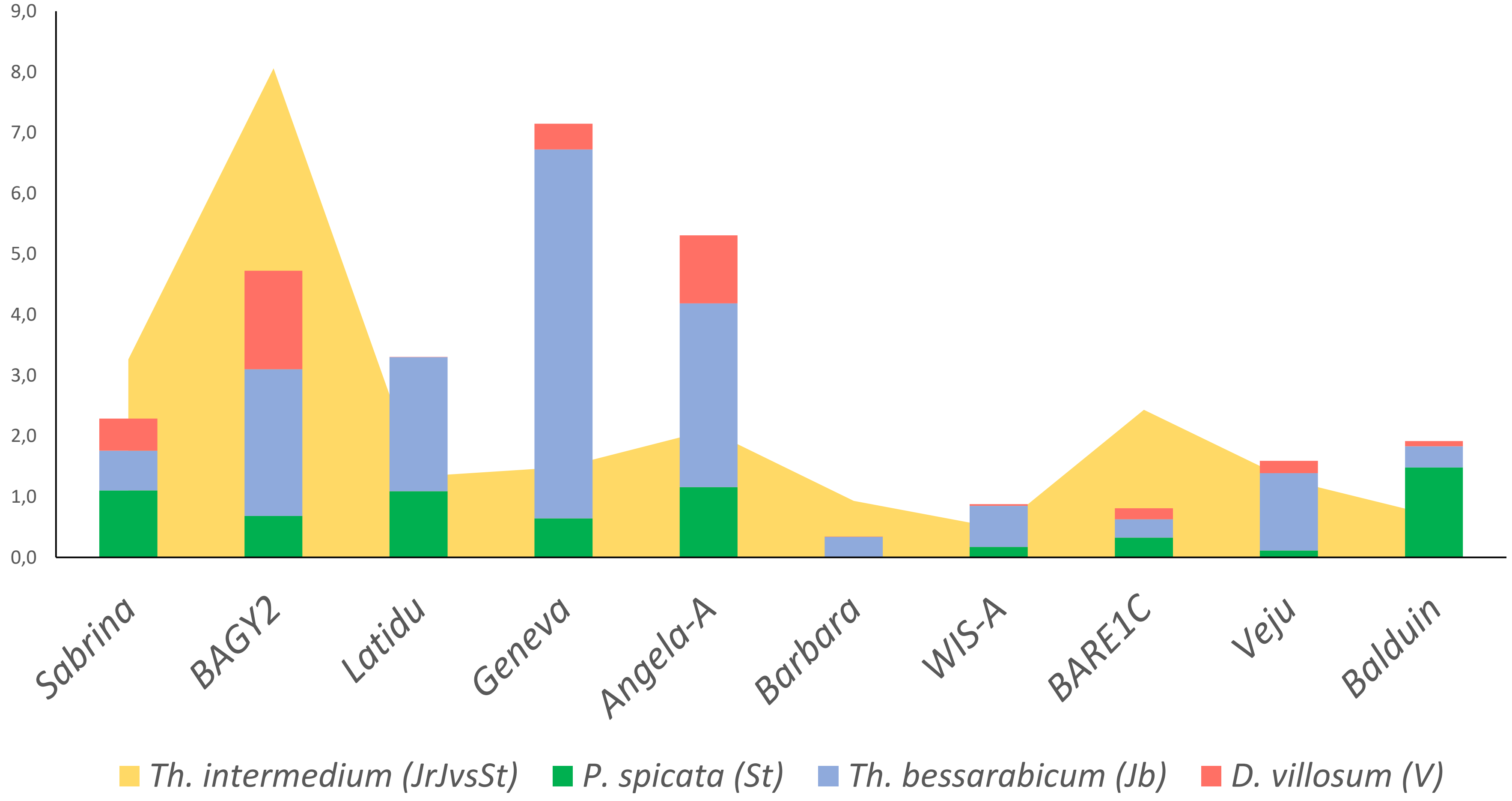

Figure S12. Normalized relative quantity (compared to *Ae. tauschii* (D), set as 1, see text) of the studied mobile elements in the following species: *P. spicata* (St), *Th. bessarabicum* (J^b), *D. villosum* (V), *Th. intermedium* ($J^rJ^{vs}St$, per whole allopolyploid genome).
