# Supplementary material for: Copy Number Variation of Transposable Elements in Thinopyrum intermedium and Its Diploid Relative Species
Source: Plants (Basel). 2019 Dec 21;9(1):15. doi: 10.3390/plants9010015 (PMC7020174; doi:10.3390/plants9010015)
Supplement: Supplementary file 1 [file plants-09-00015-s001.zip › plants-620983-Supplementary/Tables S1-S5.docx]

Copy Number Variation of Transposable Elements in *Thinopyrum intermedium* and Its Diploid Relative Species

Mikhail G. Divashuk ^1,2^, Gennady I. Karlov ^1,2^, Pavel Yu. Kroupin ^1,2,*^

^1^ Laboratory of Applied Genomics and Crop Breeding, All-Russia Research Institute of Agricultural Biotechnology, Timiryazevskaya st. 42 Moscow 127550 Russia; divashuk@gmail.com (M.G.D.), karlov@iab.ac.ru (G.I.K.), pavelkroupin1985@gmail.com (P.Yu.K.)

^2^ Centre for Molecular Biotechnology, Russian State Agrarian University-Timiryazev Agricultural Academy, Timiryazevskaya str. 49 Moscow 127550 Russia, divashuk@gmail.com (M.G.D.)

***** Correspondence: pavelkroupin1985@gmail.com

Supplementary material File 2 contains three tables: Tables S1 demonstrates the efficiency of the primers used in the qPCR experiments, Table S2 shows melting temperature for the fragments amplifies in the qPCR experiments, Tables S3 and S4 show the transposable element content in the studied species; Table S5 shows the primers used for the qPCR experiments.

**Table S1.** The efficiency of the primers used in qPCR experiments with serial dilutions of DNA template (10, 2, 0.4 and 0.08 ng per 25 μl of PCR mix) for each primer pair as calculated using LightCycler96 software.

| Transposable element | | *P. spicata* | *Th. bessarabicum* | *D. villosum* | *Th. intermedium* | *Ae.tauchii* |
| --- | --- | --- | --- | --- | --- | --- |
| *Ty3/Gypsy* | *Sabrina* | 2.05 | 2.07 | 2.05 | 2.14 | 2.01 |
|  | *BAGY2* | 2.09 | 1.96 | 2.02 | 2.07 | 1.99 |
|  | *Latidu* | 2.03 | 1.88 | 2.04 | 2.06 | 1.99 |
|  | *Geneva* | 2.05 | 1.88 | 1.93 | 2.05 | 1.94 |
| *Ty1/Copia* | *Angela-A* | 2.03 | 1.95 | 1.96 | 2.08 | 2.02 |
|  | *Barbara* | 2.02 | 1.96 | 1.97 | 2.02 | 1.94 |
|  | *WIS-A* | 2.02 | 1.90 | 1.96 | 1.99 | 1.85 |
|  | *BARE1C* | 2.03 | 1.87 | 1.91 | 1.88 | 1.87 |
|  | *Veju* | 1.96 | 1.91 | 1.93 | 1.99 | 1.93 |
| TIR | *Balduin* | 2.01 | 1.92 | 1.93 | 2.04 | 1.94 |
| Reference gene | *VRN1* | 2.03 | 1.95 | 1.92 | 2.02 | 1.98 |

**Table S2.** Melting temperature of the fragments amplified in qPCR in *P. spicata*, *Th. bessarabicum*, *D. villosum*, *Th. intermedium* (°C).

| Transposable element | | *P. spicata* | *Th. bessarabicum* | *D. villosum* | *Th. intermedium* | *Ae.tauchii* |
| --- | --- | --- | --- | --- | --- | --- |
| *Ty3/Gypsy* | *Sabrina* | 78 | 78 | 78 | 78 | 78 |
|  | *BAGY2* | 85 | 85 | 86 | 85 | 85 |
|  | *Latidu* | 83 | 83 | 83 | 83 | 83 |
|  | *Geneva* | 83,5 | 83,5 | 84 | 84 | 84 |
| *Ty1/Copia* | *Angela-A* | 80,5 | 80,5 | 80,5 | 80,5 | 80 |
|  | *Barbara* | 82,5 | 82,5 | 82,5 | 82,5 | 82,5 |
|  | *WIS-A* | 81 | 83 | 81,5 | 83 | 82 |
|  | *BARE1C* | 84,5 | 84,5 | 84,5 | 84,5 | 84,5 |
|  | *Veju* | 86 | 86 | 86 | 86 | 85,5 |
| TIR | *Balduin* | 82 | 82 | 82 | 82 | 82,5 |
| Reference gene | *VRN1* | 82,5 | 81 | 82,5 | 81 | 82,5 |

**Table S3.** The relative quantity (RQ) ± standard error of nine LTR retrotransposons and one TIR transposon in *P. spicata*, *Th. bessarabicum*, *D. villosum*, *Th. intermedium* (per one average subgenome), *Th. ponticum*. The RQ is calculated per one average genome of each species

| Transposable element | | *P. spicata* (St) | *Th. bessarabicum* (J^b^) | *D. villosum* (V) | *Th. intermedium* (J^r^J^vs^St) |
| --- | --- | --- | --- | --- | --- |
| *Ty3/Gypsy* | ***Sabrina*** | 8428±1526 | 4506±55 | 4021±836 | 7484±2086 |
|  | ***BAGY2*** | 318±58 | 1148±36 | 756±144 | 1276±353 |
|  | ***Latidu*** | 20±4 | 35±1 | 0.02±0.007 | 7±2 |
|  | ***Geneva*** | 7±1 | 67±4 | 5±1 | 5±1 |
| *Ty1/Copia* | ***Angela-A*** | 2353±475 | 8345±302 | 2271±507 | 1928±556 |
|  | ***Barbara*** | 3±1 | 490±13 | 0.5±0.1 | 454±124 |
|  | ***WIS-A*** | 2129±665 | 8458±172 | 363±69 | 2002±550 |
|  | ***BARE1C*** | 152±27 | 159±5 | 85±16 | 429±117 |
|  | ***Veju*** | 2±0.5 | 44±2 | 4±1 | 15±4 |
| TIR | ***Balduin*** | 1387±306 | 391±16 | 78±15 | 258±71 |

**Table S4.** Normalized relative quantity (NRQ, compared to *Ae*. *taushii*, set as 1) ± standard deviation of nine LTR retrotransposons and one TIR transposon in *P. spicata*, *Th. bessarabicum*, *D. villosum*, *Th. intermedium*. In *Th. intermedium*, NRQ is calculated per one average subgenome (×1) and per total genome (×3).

| Species | | *Ty3/Gypsy* | | | | *Ty1/Copia* | | | | | TIR |
| --- | --- | --- | --- | --- | --- | --- | --- | --- | --- | --- | --- |
|  |  | ***Sabrina*** | ***BAGY2*** | ***Latidu*** | ***Geneva*** | ***Angela-A*** | ***Barbara*** | ***WIS-A*** | ***BARE1C*** | ***Veju*** | ***Balduin*** |
| *P. spicata* (St) | | 1.10±0.07 | 0.68±0.09 | 1.09±0.10 | 0.64±0.14 | 1.16±0.07 | 0.003±0.001 | 0.17±0.12 | 0.33±0.10 | 0.11±0.01 | 1.48±0.22 |
| *Th. bessarabicum* (J^b^) | | 0.66±0.02 | 2.42±0.08 | 2.21±0.03 | 6.08±0.22 | 3.03±0.08 | 0.33±0.02 | 0.68±0.03 | 0.30±0.02 | 1.27±0.06 | 0.35±0.02 |
| *D. villosum* (V) | | 0.53±0.26 | 1.62±0.64 | 0.0012±0.0017 | 0.42±0.10 | 1.12±0.25 | 0.001±0.0003 | 0.03±0.03 | 0.18±0.09 | 0.21±0.09 | 0.08±0.02 |
| *Th. intermedium* (J^r^J^vs^St) | ×1 | 1.09±0.32 | 2.69±0.62 | 0.45±0.12 | 0.50±0.23 | 0.70±0.13 | 0.31±0.07 | 0.16±0.06 | 0.81±0.10 | 0.43±0.10 | 0.23±0.06 |
|  | ×3 | 3.3 | 8.1 | 1.3 | 1.5 | 2.1 | 0.9 | 0.5 | 2.4 | 1.3 | 0.7 |

**Table S5.** Primer sequences for the transposable elements and reference gene *VRN1* (Yaakov et al., 2013; Kraitshtein et al., 2010).

|  |  |  |  |
| --- | --- | --- | --- |
| *Ty3/Gypsy* | *Sabrina* | F | 5' CAT TTT CAT AGC CAT TCC GAG AT 3' |
|  |  | R | 5' CGG AAC GGT GGT AGT TGC AT 3' |
|  | *BAGY2* | F | 5' AAG AAG АТС GGA AGC GTG GA 3' |
|  |  | R | 5' АТС ATG TGG TTG GCG TTG AG 3' |
|  | *Latidu* | F | 5' CCG CTC ATG GTG TTG GAG T 3' |
|  |  | R | 5' GAG CCG CAT CGT GGA CTG 3' |
|  | *Geneva* | F | 5' TGC ATG AAG AAA AAT CGC CA 3' |
|  |  | R | 5' GAC ATG CCC CAT GCA CAT AC 3' |
| *Ty1/Copia* | *Angela-A* | F | 5' TGA ATG TTG CAG ACC CGT TG 3' |
|  |  | R | 5' GAT CAT GTT TTG CTC GTG GAA G 3' |
|  | *Barbara* | F | 5' GAC ATA ACC CTG CCG TTT GG 3' |
|  |  | R | 5' СTT CAC GGT TCG GTT CTC TCC 3' |
|  | *WIS-A* | F | 5' GAG ACA GGT TCC ACC GAC TGA 3' |
|  |  | R | 5' CCG CTA GCC ACC TTA TGC AA 3' |
|  | *BARE1C* | F | 5' ACG TGA GAC TGG TTC CAC CG 3' |
|  |  | R | 5' CCT TTT CAT CAA ATC CAA TCC G 3' |
|  | *Veju* | F | 5' TCG AGT CTC AAG GGT CGC A 3' |
|  |  | R | 5' TGG TCT GAT GGA AGC GTG AA 3' |
| TIR | *Balduin* | F | 5' GAG AAT GCT ATG ACG CAA TGA TG 3' |
|  |  | R | 5' TGG TAC AGG TTT GCA GGC ATA A 3' |
| Reference gene | *VRN1* | F | 5' TGG GAG AGG ATC TTG AAT CTT TG 3' |
|  |  | R | 5' GAT ATG TTT CAG TGA GCT TTC CAG C 3' |
